# Neuron synchronization analyzed through spatial-temporal attention

**DOI:** 10.3389/fncom.2025.1655462

**Published:** 2025-10-16

**Authors:** Haoming Yang, Pramod KC, Panyu Chen, Hong Lei, Simon Sponberg, Vahid Tarokh, Jeffrey A. Riffell

**Affiliations:** ^1^Department of Electrical and Computer Engineering, Duke University, Durham, NC, United States; ^2^Department of Biology, University of Washington, Seattle, WA, United States; ^3^Department of Computer Science, Duke University, Durham, NC, United States; ^4^School of Life Sciences, Arizona State University, Tempe, AZ, United States; ^5^Schools of Physics and Biological Sciences, Georgia Institute of Technology, Atlanta, GA, United States

**Keywords:** neural synchronization, bio-inspired neural networks, generative model, attention-mechanism, antennal lobe

## Abstract

Neuronal synchronization refers to the temporal coordination of activity across populations of neurons, a process that underlies coherent information processing, supports the encoding of diverse sensory stimuli, and facilitates adaptive behavior in dynamic environments. Previous studies of synchronization have predominantly emphasized rate coding and pairwise interactions between neurons, which have provided valuable insights into emergent network phenomena but remain insufficient for capturing the full complexity of temporal dynamics in spike trains, particularly the interspike interval. To address this limitation, we performed *in vivo* neural ensemble recording in the primary olfactory center—the antennal lobe (AL) of the hawk moth *Manduca sexta*—by stimulating with floral odor blends and systematically varying the concentration of an individual odorant within one of the mixtures. We then applied machine learning methods integrating modern attention mechanisms and generative normalizing flows, enabling the extraction of semi-interpretable attention weights that characterize dynamic neuronal interactions. These learned weights not only recapitulated the established principles of neuronal synchronization but also facilitated the functional classification of two major cell types in the antennal lobe (AL) [local interneurons (LNs) and projection neurons (PNs)]. Furthermore, by experimentally manipulating the excitation/inhibition balance within the circuit, our approach revealed the relationships between synchronization strength and odorant composition, providing new insight into the principles by which olfactory networks encode and integrate complex sensory inputs.

## 1 Introduction

Interconnected neural populations construct a meaningful perception of the sensory features of the complex external world (Hopfield, 1995; Reiter and Stopfer, 2013). Neural representations of the sensory world are temporally structured, and this temporal organization drives the selective behaviors by influencing which neurons are recruited, when they are activated, and how intensely they fire in the central brain (Hopfield, 1995; Laurent, 2002). The coordinated timing of neural activity of these neurons—referred to as synchronization—is thought to enhance sensory perception, sharpen neural representation, and enable complex sensory discrimination. This allows the foraging species to exhibit fast and effective decisions by facilitating effective transmission of information to the downstream targets (Riffell et al., 2009a, 2014).

Sensory signals are dynamic and multidimensional, requiring the brain to integrate the information across both time and space through distributed neuronal populations to generate meaningful representations (Laurent, 2002; Wachowiak et al., 2025). These spatial-temporal properties of the neurons necessitate the synchronization among neurons to encode identity, intensity, and valence of different sensory stimuli, including odors (Stopfer et al., 2003; Bitzenhofer et al., 2022). Odor processing involves the synchronization of the neural activity across multiple levels of the brain. In mammals, neural synchrony occurs in the olfactory bulb (OB). It may drive activity through sets of mitral and tufted cells in higher brain centers, such as the piriform cortex, entorhinal cortex, and amygdala (Laurent, 2002; Wilson and Sullivan, 2011). Similar processes may be at play in insects, where neural synchrony occurs in the primary olfactory center, the antennal lobe (AL), and activates downstream neurons in the higher brain centers, including the lateral horn and mushroom body, thought to be involved in valence and learning and memory, respectively (Riffell et al., 2009b). The synchronized activities of neurons in higher brain centers are represented not only by firing rate but also by the precise timing of neuronal activity among tens of thousands of neurons. The interaction between these neurons remains an open question (Laurent, 2002; Stopfer et al., 1997).

The insect olfactory system provides a tractable neuroanatomical model for examining the functional bases of olfactory processing. Olfactory detection starts with the activation of the olfactory sensory neurons, and axons of these neurons terminate in the distinct anatomical structures called glomeruli in the primary olfactory center, the antennal lobe (Chu et al., 2020; Kymre et al., 2021; Lei et al., 2011). Each glomerulus is innervated by several projection neurons (PNs) that relay information from the AL to the higher brain areas (Homberg et al., 1988; Kymre et al., 2021). The odor-activated PNs interact with each other via a dense network of inhibitory neurons, local interneurons (LNs) spanning throughout the AL (Hoskins et al., 1986). Several studies have shown that odorants evoke strong synchronous firing of the PNs in several species (Christensen et al., 1987; Heinbockel et al., 2004; Lei et al., 2002; Martin et al., 2013; Nagel and Wilson, 2016; Tsai et al., 2018; Wilson et al., 2004), providing odor�specific representations and encoding (Mazor and Laurent, 2005; Stopfer et al., 1997). However, studies have typically used monomolecular odorants or binary mixtures, which do not represent the complexity of the natural olfactory environment (Riffell et al., 2009a; Wachowiak et al., 2025).

Natural odors are complex mixtures made up of tens to hundreds of compounds (Bisch-Knaden et al., 2022; Riffell et al., 2009a). The proportion of odorants in the mixture is critical in odor encoding, particularly in a dynamic environment. Previous studies have shown that changing the concentration of only one compound significantly affects an insect's ability to discriminate and track the floral odor (Riffell et al., 2009b). However, the cellular and computational bases by which the olfactory system binds specific features of the complex odor mixture—including the critical odorants—are not known, and neuromorphic principles that are involved in such processes are still uncertain, given the diverse physiological and morphological properties of these neuron types (Biederlack et al., 2006; Carlsson et al., 2007; Guerrieri et al., 2005).

Despite advances in modeling either spatial or temporal neural dynamic patterns, current methods fall short in jointly capturing spatiotemporal dynamics due to computational and structural limitations. Previous methods for temporal [recurrent neural network (Laurent et al., 1996), Hebbian plasticity (Delahunt et al., 2018)] or spatial aspects of neural responses have been investigated. In these established methods, either a paired or a subset of the neurons (Stopfer et al., 2003) were commonly utilized in investigating the temporal dynamics, but the interaction of the neurons at the population level has been broadly overlooked. Generally, the correlation between neurons is a common strategy to investigate the sensory encoding in population codes based on the tuning similarities, stimulus effects, and the presence of higher order correlations (reviewed in Panzeri et al., 2022). In previous studies, responsive neurons were considered for investigation, whereas non-responsive units, often excluded from analysis, are frequently encountered during experiments (Fiscella et al., 2015; Ni et al., 2022; Pasupathy and Connor, 2002). However, the non-responsive units could play an important role in population coding through synergy with the responsive neurons (Haggard and Chacron, 2025). The mechanism by which individual neurons in a population selectively weigh the stimulus and influence other neurons has not been investigated.

In this study, we address how an olfactory neural population encodes complex odor information for decision making. Through *in vivo* neural ensemble recordings and computational analyses, we treat the spiking of neurons as a point process in time. Instead of assuming the arrival time of spikes follows a canonical distribution (e.g., Poisson distribution), we use the highly flexible, non-parametric, deep normalizing flow to model the probability distribution of interspike intervals (ISIs: Kobyzev et al., 2021). During the modeling process of the spike train of a specific neuron in the AL, we introduce a novel spatial-temporal attention module to learn how individual neurons synchronize with the rest of the neuron population (spatial) and are affected by population spike trains dynamically (temporal). This spatial attention weight module accounts for the higher order interactions across a population of neurons, allowing us to analyze complex population-level synchronization beyond the pairwise analyses of Ensemble Synchronization Index (Riffell et al., 2009b) and Kernelized binless methods (Martin et al., 2013). We found that our method outperforms in the classification of odors, considering both the interactions within and between LNs and PNs in the AL. In addition, our study indicates that increasing the proportion of a compound in the mixture—thereby altering the excitation/inhibition balance in the AL—could reduce the pattern of neural synchrony.

## 2 Data curation

### 2.1 Insect preparation

Adult male moths (*Manduca sexta*; Lepidoptera: Sphingidae) were reared in the laboratory on an artificial diet under a long-day (17/7-h light/dark cycle) photoperiod. The moths (3 days old, post-eclosion) were secured in a 10 ml plastic pipette (Thermofisher Scientific, USA) with dental wax (Kerr Corporation, Romulus, MI, USA), leaving the head and antennae exposed. The cuticle on the head was carefully cut to expose the brain, and all the muscles, trachea, and neural sheath were carefully removed with fine forceps (Fine Science Tool, USA). The restrained moth was mounted to a recording platform attached to the vibration isolation table. The preparation was placed such that the ALs are orientated dorsofrontally. The brain was superfused slowly with physiological saline solution [150 mM NaCl, 3 mM CaCL_2_, 3 mM KCl, 10 mM N-Tris(hydroxymethyl) methyl-2 aminoethanesulfonic acid buffer, and 25 mM sucrose, pH 6.9] throughout the experiment.

### 2.2 Odor stimulation

**Figure 1:**
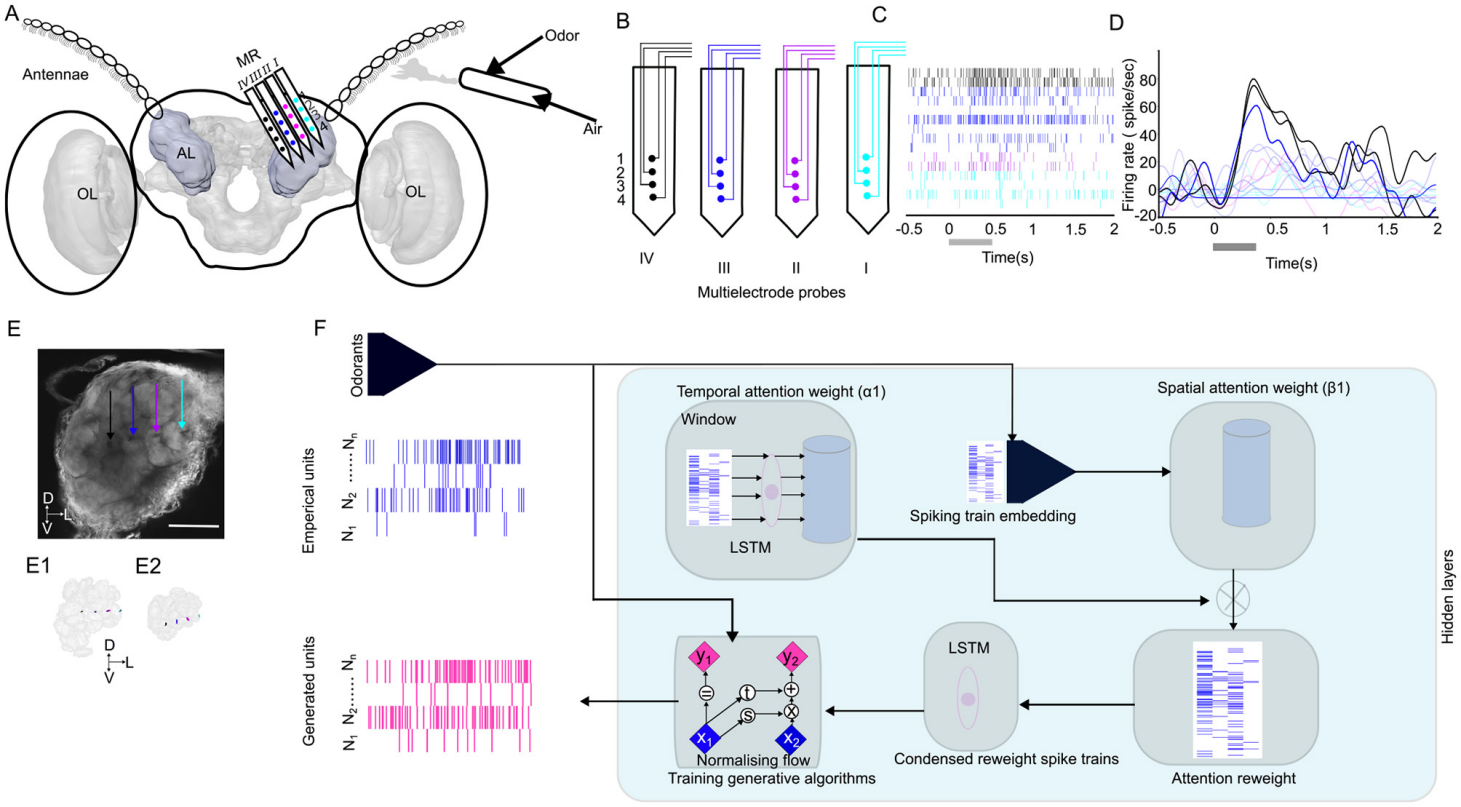
Overview of methods applied in this study. **(A)** Schematic of the insertion of the 16-channel multielectrode recording array (MR; 4 sites × 4 shanks) in the antennal lobe (AL) of the male moth, where the odor is delivered on the antennae. The three-dimensional (3D) model of the male moth brain is acquired from (https://www.insectbraindb.org; Heinze et al., 2021). Images are not to scale. **(B)** Each site on a given shank is assigned the same color to indicate tetrode grouping during spike sorting. **(C)** A representative spike raster plot of the units, color-coded with multielectrode probes stimulated with B_23_. The first trial of the stimulation was presented out of the five trials. The gray bar represents the stimulation duration of 400 ms. **(D)** Peristimulus time histograms, superimposed by different units. The darker line indicates the stronger response, and the lighter color indicates weaker responses. **(E)** Maximum-intensity projection of the AL demonstrates the location of the probes within the AL. The scale bar is 100 μm. Each shank is represented with the arrows corresponding to the channel color in B. The 3D reconstruction of the probes and glomerulus that is impeded by these probes in the AL in dorsal **(E1)** and lateral **(E2)** orientations. **(F)** Architecture of the spatial-temporal attention normalizing flow. The spike trains are first passed through a Long Short-Term Memory (LSTM) unit and linear embedding modules to obtain the spatial-temporal attention weights for reweighting the spike train. The reweighted spike train is then passed through a second LSTM module, and its final hidden representation of the reweighted spike train is used as the context vector to train the conditional normalizing flow for learning distributions of the interspike intervals (ISIs) and generate realistic spike trains. The *x, y* in the Normalizing flow denotes the input and output of an affine coupling layer (Dinh et al., 2016); the subscripts 1, 2 denote different parts of the latent variable; *t* and s denote two different neural networks. OL, optic lobe; D, dorsal; L, lateral; V, ventral.

**Figure 2:**
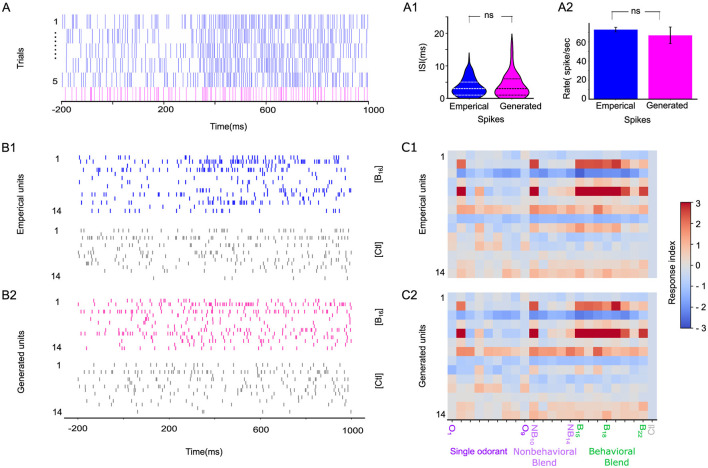
STAN-flow learns the generating distribution of interspike interval (ISI). **(A)** A representative odor-evoked spike train of a particular neuron across five trials that are empirically generated (blue). The spike train generated by the model for the same neuron (magenta) is placed on the lower panel. The gray bar indicates the stimulation window of odor blend (B_19_); the generated spike train is sampled by conditioning on the test set during cross-validation. **(A1)** Violin plot of ISI of empirical and generated spike train in **(A)** under B_19_ stimuli, the dashed line indicates the 25%, 50%, and 75% quartiles. **(A2)** Comparison of the average firing rate of empirical vs. generated spikes. **(B1)** Empirical spike trains of 14 neurons stimulated with B_16_ (blue) and control (gray). **(B2)** Generated spike trains of the same neuron stimulated with B_16_ (magenta) and control (gray); the gray bar indicates the stimulation window. **(C1)** Response Index of the empirical recordings of population of neural responses of the same preparation in B and the model-generated neural responses **(C2)** to the single odorants (O_1_–O_9_; purple), non-behavioral blends (NB_10_–NB_14_; light purple), and behaviorally relevant blends (B_15_–B_22_; green). ns, not significant.

Pulses of air (100 ml/min) were pushed through a glass cartridge containing a piece of Whatman filter paper (Millipore Sigma, USA) loaded with 10 ul of floral odorant and injected into a constant air stream (1 L/min) directed toward the moth's antennae (Figure 1A). The stimulus was pulsed through a solenoid-actuated valve controlled by an RZ2 bioamplifier processor (Tucker-Davis Technologies, Inc., FL, USA). The outlet of the stimulus cartridge was positioned 2 cm from and orthogonal to the center of the antennal flagellum. Stimulus duration was 400 ms, and five pulses were separated by either a 5-s interval or a 10-s interval. The interstimulation duration was approximately 1 min. The tested stimuli were categorized as behavioral (B, green colored) and non-behavioral (purple and light purple colored; Figures 2C1, C2, 3A–C, 4C, D, 5C–F). Different odor stimuli under each category are annotated with the subscripted number. We classify the blend as a behavioral one if it contains all three compounds: Benzaldehyde (O_1_), Benzyl alcohol (O_2_), and Linalool (O_6_). For our first sets of an experiment, we tested behavioral stimuli including extracts of Datura flowers (B_15_), five artificial mixtures (B_16_–B_19_) containing the behavioral components, three dilutions of B_19_ [10 times dilution(B_20_), 100 times dilution (B_22_), 10,000 times dilution (B_22_)] and non-behavioral stimuli include mineral oil (control, no odor), five mixtures of non-behavioral components (NB_10_ - NB_14_), and nine individual non-behavioral components (O_1_–O_9_). In the second experiment, to determine how modifying the ratio of compounds in the mixture modified the encoding of floral odor, we used an odor cartridge containing the behavioral mixture (B_23_) and a second odor cartridge containing increased concentrations of Benzaldehyde (O_1_) with 10-, 100- or 1,000-fold higher concentrations (B_24_, B_25_, and B_26_). The odors from the two odor cartridges were released simultaneously into the airway, allowing them to mix before reaching the flagellum. In this manner, the ratio of compounds in the behavioral mixture (B_23_) could be dynamically altered. See Table 1 for the stimuli and their compositions.

**Table 1:** Odor stimulation and the mixture composition.

**Stimuli class**	**Stimuli**	**Name**	**Composition**
**Single odorants**	**O_1_**	**Benzaldehyde**	
	O_2_	Benzyl alcohol	
	O_3_	Farnesene	
	O_4_	Geraniol	
	O_5_	Caryophyllene	
	O_6_	(±)Linalool	
	O_7_	Methyl salicylate	
	O_8_	Myrcene	
	O_9_	Nerol	
Non-behavioral blend	NB_10_		Benzaldehyde + Benzyl alcohol
	NB_11_		Methyl salicylate + Caryophyllen + Farnesene
	NB_12_		Methyl salicylate + Caryophyllen + Farnesene+Myrcene
	NB_13_		Methyl salicylate + Caryophyllen + Farnesene + Myrcene + Geranoil
	NB_14_		Methyl salicylate + Caryophyllen + Farnesene+ Myrcene + Geranoil + Nerol
Behavioral blend	B_15_	Datura extract	
	B_16_		Benzaldehyde + benzyl alcohol + linalool
	B_17_		Benzaldehyde + benzyl alcohol + linalool+nerol
	B_18_		Benzaldehyde + benzyl alcohol + linalool+ nerol + geraniol
	B_19_		Benzaldehyde + benzyl alcohol + linalool + nerol + geraniol + myrcene + methyl salicylate + caryophyllene + farnesene
	B_20_	10 × dilution of B19	
	B_22_	100 × dilution of B19	
	B_22_	10,000 × dilution of B19	
	B_23_		Benzaldehyde + benzyl alcohol + linalool + nerol+geraniol + methylsalicylate + methylbenzoate
	B_24_	10 × increase of Benzaldehyde	
	B_25_	100 × increase of Benzaldehyde	
	B_26_	1000 × increase of Benzaldehyde	
Control	Ctl	Mineral oil	

**Figure 3:**
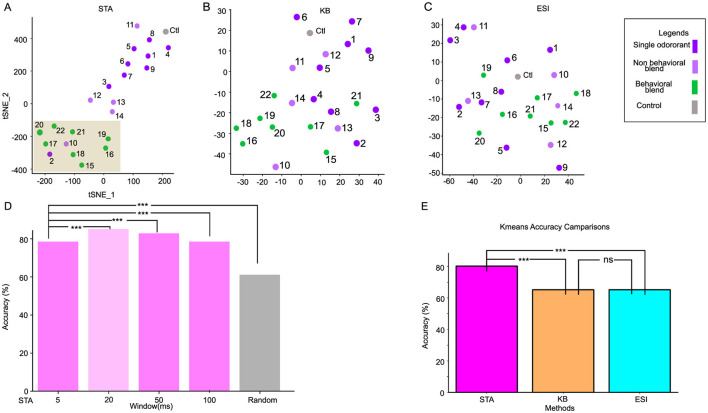
Clustering behavioral and non-behavioral stimuli through different synchronization methods. **(A)** spatial-attention weights separate the behavioral stimuli (green) from the non-behavioral stimuli (purple) by forming two distinctive clusters in the 2D TSNE-reduced space. The behavioral stimuli cluster is highlighted with a brown rectangle. **(B, C)** 2D TSNE plot of Binless synchronization (KB) and Ensemble Synchronization Index (ESI), respectively. **(D)** The bargraph represents the accuracy of the 2-class K-means algorithm across various window sizes. The window used in our analysis is shown against the light green background. While STA(20ms) is significantly better than other window sizes, the STA generally outperforms traditional methods. **(E)** The bargraph represents the 2-class K-means algorithm with the spatial-temporal attention (STA) method, repeated with 100 different initializations. K-means accuracies of the Kernel Binless (KB) and Ensemble Synchronization index (ESI) are significantly less accurate than STA. ^***^p-value < 0.001 and ns is non-significant.

**Figure 4:**
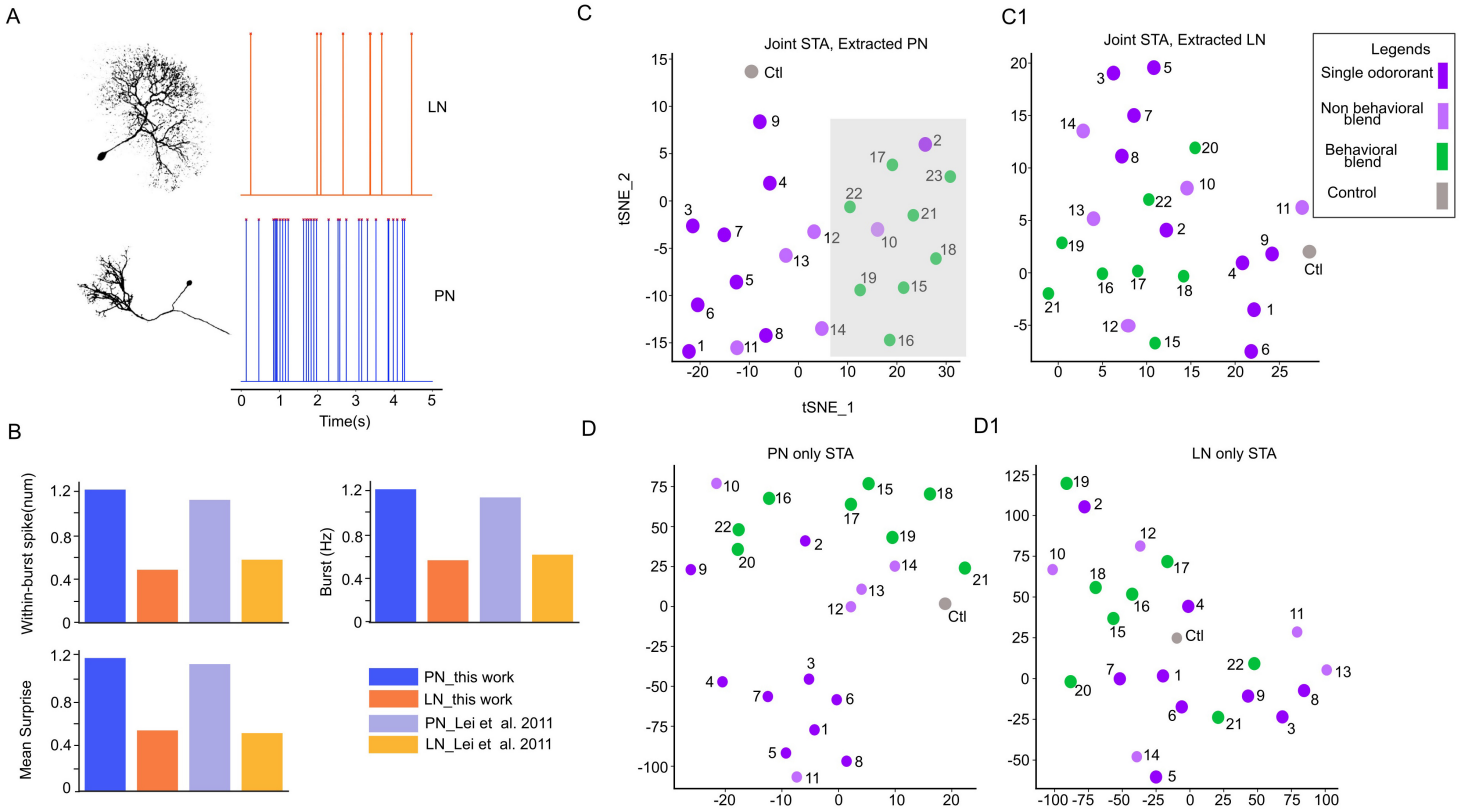
Spatial-attention module learns the interaction between two neuron populations in the antennal lobe. **(A)** Representative morphology and physiological features of the local interneurons (LNs) and Projection neurons (PNs). LN spikes more regularly while PN spikes in a burst pattern. **(B)** Selected electrophysiology features to classify neuron types; the difference between PN and LN is consistent with previous analysis of (Lei et al. 2011). **(C)** The spatial-attention of PNs and LNs **(C1)** was separately extracted from a joint model (trained with both PNs and LNs) and then reduced to two dimensions through TSNE. The behavioral cluster of stimuli is highlighted with a light-shaded gray rectangle. **(D)** TSNE reduced two-dimensional (2D) scatter plot of spatial-attention with the model trained with PNs only and with LNs only **(D1)**.

**Figure 5:**
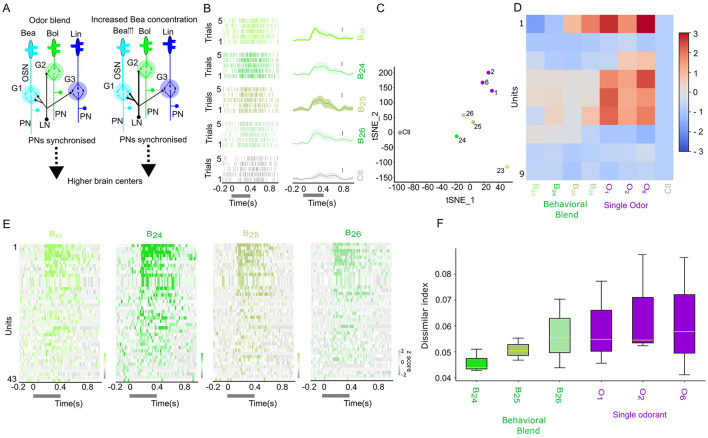
Changes in synchronization pattern with increased Benzaldehyde (O_1_) ratio in behavioral mixture. **(A)** Hypothetical neural circuits mediating the role of local inhibition by the Local interneurons (LN, black color) to the olfactory sensory neurons (OSNs) relaying three different floral odors (Benzaldehye (O_1_), Benzylalcohol(O_2_), and Linalool(O_6_) at naturalistic odor blend and floral odor with increased O_1_ ratio. The arrowhead besides O_1_ indicates the increased concentration. The PNs are synchronized, and olfactory information is relayed from the AL to the higher brain centers. The thicker line of the LNs, when presented with increased O_1_ ratio, could indicate the involvement of the modulatory effect. **(B)** Odor-evoked activity of a single neuron to the increased (O_1_) ratio in the behavioral mixture for five trials (top row: natural behavior concentration (B_23_), second row (10 × increase in (O_1_) ratio–(B_24_)), third row (100 × increase in (O_1_)–(B_24_)), and fourth row (1,000 × increase in (O_1_)–(B_25_)). The bottom row represents the control (Ctl). Spike raster plot, right side; peristimulus histogram with light shaded present mean±sem, left side. The vertical bar represents the firing rate of 20 spikes per second. **(C)** Two-dimensional scatterplot of the TSNE reduced spatial-attention weights. **(D)** Response index of the generated spike showing B_23_ odor, with increased ratio of O_1_ and individual odorants. **(E)** *z*-scored odor-evoked activity of 43 neurons from 3 moths of B_23_–B_26_. **(F)** Dissimilar index of odor B_23_ computed against different ratios of increased O_1_ in B_23_ and individual odorant.

### 2.3 Ensemble antennal lobe recording

Odor-evoked responses were obtained from 80 units in seven male moths. Recordings were made with 16-channel silicon multielectrode recording (MR) arrays (A4X4-3 mm-50-177; NeuroNexus Technologies). These probes have four shanks (each of 15 μm in thickness) spaced 125 μm apart, each with four recording sites 50 um apart, and have a surface area of 177 μm^2^ indicating the distinct spatial activity patterns across the different regions in the antennal lobe (Figures 1B–E) (Lei et al., 2004). The MR was positioned under visual control with a stereo microscope (Narishige, Japan). As demonstrated in Figures 1A, E, the four shanks were oriented in a line parallel to the antennal nerve. The MR was advanced slowly through the AL with a micromanipulator (Narishige, Japan) until the uppermost recording sites were just below the surface of the AL. Thus, the four shanks of the MR were recorded from four regions of glomerular neuropil across the AL. Ensemble activity was recorded simultaneously from the 16 channels of the MR array by using TDT amplifiers (Tucker-Davis Technologies, Inc., FL, USA). The recorded signal was digitized at 25 kHz per channel by using Synapse software (version 98, Tucker-Davis Technologies, Inc., FL, USA).

### 2.4 Localization of recording probes in the AL

The head was excised, and the brains were dissected in Manduca saline. The brain was washed with 0.01 M phosphate-buffered saline (PBS) (2 times: each for 20 min) and then submerged in the solution consisting of 4% PFA and 0.03% glutaraldehyde) to facilitate tissue fixation. The preparation was kept overnight at 4°C and then dehydrated in a series of ethanol (50%, 70%, 90%, 96%, 100%, and 100%: each for 20 min). Finally, it was cleared in methyl salicylate (Millipore Sigma, USA). The whole-mount preparation is scanned with a laser scanning microscope (Nikon, A1R, Nikon Instruments, Inc., USA) equipped with a CFI Plan Apo 10 × Air objective, which is scanned with a 488 nm line of an argon laser. The high-resolution confocal images with 1,024 × 1,024 pixels at a distance of 2–4 μm in the *z*-direction were obtained. The image was imported in AMIRA version 6.5.0 (Thermofisher Scientific, USA) and the glomerular structures were reconstructed (Figure 1E). The shank impaled in the AL was also reconstructed and visualized.

### 2.5 Spike sorting

The continuous waveforms are exported to an offline sorter (Offline sorter, Plexon, Version 4.7.1). The spike data were digitized at 25 kHz per channel. The filter setting (0.6–5 kHz and system gain of 1,000 were software adjustable on each channel. Spikes were sorted by tetrode configuration using a clustering algorithm based on the method of principal components (PCs) (Off-line Sorter; Plexon). We used dual thresholds, between 2 and 3 standard deviations (SD) above the baseline. The highest and lowest thresholds will eliminate the voltage deflections deriving from movement artifacts that are misinterpreted as spikes. The groups that were separated in the 3D space (PC1–PC3) after statistical verification (multivariate analysis of variance (ANOVA); *p* < 0.05) were selected for further analysis (7–19 units were isolated per ensemble; Figures 1C, D). Each spike in each cluster was time-stamped, and these data were used to create raster plots and calculate peristimulus time histograms (PSTHs). Preliminary analyses were performed with Neuroexplorer (Nex Technologies, version 5.4) using a bin width of 5 ms unless otherwise stated.

## 3 Model

In this section, we present Spatial-Temporal Attention Normalising Flow (STAN-Flow), a generative framework for modeling neuronal spike train dynamics through the distribution of interspike intervals (ISIs). Our approach combines a spatial-temporal attention module, which encodes both neuronal interactions and temporal spiking history, with a conditional normalizing flow that flexibly models ISI distributions without restrictive parametric assumptions. This integration enables us to capture stimulus-driven synchronization and timing patterns in a principled and interpretable manner. We then conclude the section with an introduction to the different methods and metrics we compute and compare in the results section.

### 3.1 Problem formulation and notation

We denote spike train *S*^*q*^ ∈ ℝ^*N*×*T*^ for a total of *N* neurons, *T* timesteps, and *q* ∈ 1, …, *Q* different stimuli. For the *n*-th neuron, we denote the time of the *i*-th spike timing as $t_{i}^{qn}$, hence the previous spike's timing as $t_{i-1}^{qn}$. Then the interarrival time between these two spikes is $\tau_{i}^{qn}=t_{i}^{qn}-t_{i-1}^{qn}$. The goal is to model the interarrival time distribution $\tau_{i}^{qn}$ for arbitrary spike *i*.

Let [Δ] denote the window size and denote a windowed spike train as *S*_*i*[Δ]_; we assume the interspike intervals of neuron *n* are conditionally independent given the stimuli *q*, the Δ-windowed history of last spike, and time of the last spike to reduce the problem into modeling the distribution of interarrival time presented in Equation 1. For ease of notation, we drop the superscript *q* and focus on an arbitrary stimulus; we also drop the superscript *n* and focus on an arbitrary neuron.


(1) $$\begin{array}{l} P\left(\tau_{i}|S_{i-1\left[\text{Δ}\right]},q,t_{i-1}\right) \end{array}$$

Our generative modeling approach consists of two main components: (1) the spatial-temporal attention units that encode the windowed spike history to latent space; and (2) a conditional generative model learned with a normalizing flow that models the target distribution presented in Equation 1. Beyond the goal of learning the conditional ISI distribution, the attention weights learned by this conditional generative system can be extracted for further analysis. We present the full spatial-temporal attention normalizing flow (STAN-Flow) architecture in Figure 1F.

### 3.2 Spatial-temporal attention

The spatial-temporal attention mechanism combines the vision attention mechanism in computer vision (Dosovitskiy et al., 2021) and temporal attention in natural language processing (Bahdanau et al., 2016; Vaswani et al., 2023). Synchronization can be viewed as interactions between neurons, which can be characterized through spatial attention, where a higher spatial attention weight corresponds to a stronger interaction between neurons. The importance of particular spike timing and the general spiking rate is characterized by the temporal weights that scan through the spiking history: the higher the temporal weight, the more important a specific time is. Therefore, neurons can be synchronized in their activity even if they have different individual temporal dynamics.

The spatial-temporal attention module consists of Long Short-Term Memory (LSTM) layers and a few linear layers. The LSTM layers effectively summarize spike train time series into lower dimensional hidden states, which are then projected by the linear layers to obtain semi-interpretable attention weights. The windowed spike train *S*_*i*−1[Δ]_ is passed through the first LSTM (*f*_1_) and a linear spatial-embedding layer parameterized as $W_{e}$ and *b*_*e*_, outputs the hidden representation *h*_*i*−1_ and *d*-dimensional spike train spatial embedding $E_{i}\in {\mathbb{R}}^{N\times d}$. For the temporal attention, we further reduce *h*_*i*−1_ through another linear layer parameterized as $W_{\alpha }$ and *b*_α_ to obtain the temporal weights, $\alpha_{i}^{t}\in {\mathbb{R}}^{\left[\text{Δ}\right]}$.


$$\begin{array}{c} E_{i}=W_{e}(S_{i-1[\text{Δ}]})+b_{e}={\left[e_{i}^{1},\ldots ,e_{i}^{N}\right]}^{\text{T}}\\ h_{i-1}=f_{1}(S_{i-1[\text{Δ}]})\\ \alpha_{i}^{t}=\text{softmax}(\text{Tanh}(W_{\alpha }h_{i-1}+b_{\alpha })) \end{array}$$

Additionally, (Riffell et al. 2009a) shows that stimuli information is also encoded by the ensemble firing of neurons. Hence we concatenate the last hidden states of the LSTM, denoted as h^*^, the spatial embedding of a particular neuron's activity $e_{i}^{m},m\in 1\ldots N$, and the stimuli *q* to pass through a linear layer parameterized as $W_{\beta }$ and *b*_β_ and obtain the spatial weights $\beta_{i}^{m}\in \mathbb{R}$. The vector that contains the spatial weights of all neurons is denoted as $\beta_{i}\in {\mathbb{R}}^{N}$. To isolate the higher-order ensemble patterns, we replace the traditional *softmax* of the attention mechanism with its sparsifying counterpart *sparsemax* (Martins and Astudillo, 2016) which directly projects logit values onto the simplex. Applying the *sparsemax* allows some attention weights to be reduced to zero, amplifying the effect of those synchronized neurons.


$$\begin{array}{c} \beta_{i}^{m}=\text{sparsemax}\left(\text{Tanh}\left(W_{\beta }\left[h^{*};e_{i}^{m};q\right]+b_{\beta }\right)\right)\\ \beta_{i}={\left[\beta_{i}^{1},\ldots ,\beta_{i}^{N}\right]}^{\text{T}} \end{array}$$

Usual applications of attention weights obtain a context vector through a weighted average of latent variables (Bahdanau et al., 2016; Vaswani et al., 2023). However, a weighted average of each neuron's representation dilutes the synchronization identified through the spatial attention weight as the resulting weighted representation becomes less identifiable. Hence, we reweight the windowed spike train with the mean-normalized spatial-temporal weights and feed the reweighted windowed spike train ($S_{i-1}^{\prime }\left[\text{Δ}\right]$) through a second LSTM layer (*f*_2_) to obtain the final hidden representation $h_{i-1}^{\prime }$. The symbol × denotes element-wise multiplication.


$$\begin{array}{l} S_{i-1\left[\text{Δ}\right]}^{\prime }=\beta_{i}{\left[\alpha_{i}^{t}\right]}^{T}\times S_{i-1\left[\text{Δ}\right]}\;\left(\text{Reweighting Spike Train}\right)\\ \;h_{i-1}^{\prime }=f_{2}\left(S_{i-1\left[\text{Δ}\right]}^{\prime }\right)\;\left(\text{Obtain final hidden representation}\right) \end{array}$$

The output of *f*_2_, $h_{i-1}^{\prime }$, can be seen as a context vector derived from the reweighted spike train. This context vector combines the temporal dynamic spike train, the neuron interaction, and the influence of different stimuli into a continuous representation to inform the conditional generative model.

It is worth noting that the attention mechanism introduced in this section is inspired by the attention mechanism first introduced in translation tasks, rather than the more well-known self-attention mechanism for transformer-based architectures (Bahdanau et al., 2016; Vaswani et al., 2023). This modeling choice is due to several practical concerns. First, the self-attention mechanism offers limited interpretability. The transformer architecture linearly embeds data into uninterpretable lower dimensions, then applies the attention mechanism on the value matrix; this limits the interpretation of the attention matrix compared to our formulation, which directly applies the attention weights to modify the spike trains. Second, fitting self-attention mechanism on the temporal and spatial dimensions through the transformer architecture requires two transformer modules; this will induce much more computation overhead and is less efficient. Finally, due to the size of transformer-based architecture, a large amount of data is usually required to leverage its ability to process diverse contexts; however, neural datasets generally lack this level of diversity to train a transformer without heavy regularization (Ye and Pandarinath, 2021).

### 3.3 Conditional normalizing flow

Once we learn the synchronization and timing information, we build a modeling module to accurately reflect the ISI distribution based on synchronization and temporal dynamics. While traditionally the modeling of spike trains follows the Poisson Process, this assumes the spike train is rate-coded and the ISI distribution follows an exponential distribution. These assumptions are not always realistic and constrain the modeling process. Instead of a model based on the Poisson assumption, we build a non-parametric deep generative model conditioned on the final hidden representation $h_{i-1}^{\prime }$ to learn the probability distribution of interspike intervals given the learned history.

We chose to apply a conditional normalizing flow that directly optimizes the negative log-likelihood of the density. A normalizing flow is usually defined by a transformation of a standard Gaussian distribution into a more complex distribution (Kobyzev et al., 2021). This transformation typically involves a sequence of invertible, tractable, and differentiable mappings, enabling the evaluation of a sample's value in the simple distribution or its likelihood.

We concatenate the stimuli *q*, the last hidden representation of attention-reweighted spike train $h_{i-1}^{\prime }$, and the time of last spike *t*_*i*−1_ into a context vector denoted as *x*_*i*_. We propose a normalizing flow that is conditioned on *x*_*i*_; the likelihood takes the following form, where *Z* is drawn from a conditional Gaussian distribution. Extending recent neural network architecture (Kobyzev et al., 2021; Dinh et al., 2016), a loss through log-likelihood can be written as:


(2) $$\begin{array}{l} x_{i}=\left[h_{i-1}^{\prime };q;t_{i-1}\right] \end{array}$$

(3) $$\begin{array}{l} \log P\left(\tau_{i}|x_{i}\right)=\log P\left(z_{i}|x_{i}\right)\text{det}\left|\frac{\partial z}{\partial \tau }\right|=\log P\left(f_{\theta }\left(\tau_{i},x_{i}\right)\right)\\ \;+\log\left(\text{det}\left|\frac{\partial f_{\theta }\left(\tau_{i},x_{i}\right)}{\partial \tau }\right|\right) \end{array}$$

We specifically applied the real-valued non-volume-preserving normalizing flow architecture (RealNVP) in our study (Dinh et al., 2016), where the *f*_θ_ is characterized through a series of neural networks that construct an upper triangular Jacobian, simplifying the determinant computation of the Jacobian to be the trace.


(4) $$\begin{array}{l} \log P\left(\tau_{i}|x_{i}\right)=\log P\left(f_{\theta }\left(\tau_{i},x_{i}\right)\right)+\text{Tr}\log\left(\left|\frac{\partial f_{\theta }\left(\tau_{i},x_{i}\right)}{\partial \tau }\right|\right) \end{array}$$

This framework considers the spiking history, the interaction between neurons, and the stimulus effect, and learns the ISI distribution without assuming it follows some canonical, parametric distribution. Hyperparameters related to the architecture and training process are listed in the Supplementary material.

### 3.4 Identifying synchronization

A crucial part of our analysis is establishing a higher order non-linear method to analyze neuron synchronization. We propose the spatial attention weight method and compare it with two previously reported neuron synchronization methods, the Ensemble Synchronization (ESI) proposed in (Lei et al. 2004), and the Kernelized Binless Method (KB) applied in (Martin et al. 2013); we then discuss the synchronization analysis process of our proposed spatial-attention weights method.

#### 3.4.1 Ensemble synchronization

The traditional analysis of ensemble patterns utilizes the cross-correlation coefficient between pairs of neurons (Lei et al., 2004). In particular, the synchronization index (SI%) is calculated as


(5) $$\begin{array}{l} {\text{SI\%}}_{\text{ensemble}}=\frac{{\text{[CE]}}_{\text{raw}}^{\delta }-{\text{[CE]}}_{\text{shuffle}}^{\delta }}{C_{1}\left(T\right)+C_{2}\left(T\right)}\times 100\% \end{array}$$

where [CE]_raw_ is the number of coincident events in the cross-correlogram peak centered around *t* = 0 with width δ, [CE]_shuffle_ is the number of coincident events after trial shuffling (shift predictor method) to correct for coincidences attributable to chance and an increased firing rate. The corrected correlograms were calculated by averaging four trial shifts and subtracting the result from the raw correlogram. *T* is the total response time over which spikes were counted, and *C*_1_ and *C*_2_ are the number of spikes recorded from units 1 and 2 during time *T* (Lei et al., 2004).

We calculated the ensemble SI% for all stimuli using one trial as the raw trial and corrected it by shuffling the other four trials. We applied the parameters δ = 5 and *T* = 1, 000 (ms) after the onset of the stimuli as suggested in (Riffell et al. 2009a). In the Supplementary Figure S1, we explore a variety of hyperparameters for δ and *T*.

#### 3.4.2 Kernelized binless method

A more recent method for analyzing the synchronization of neuron firing is through the kernelized binless method (Martin et al., 2013). While it remains a pairwise synchronization analysis, it applies an exponential function kernel to smooth out the spike train. Specifically, the exponential kernel is denoted *h*(*t*) = exp(−*t*/ϕ) × *u*(*t*) where *u*(*t*) is the heavy side function, ϕ is a kernel parameter to aggregate spikes over time; a similarity index (see Equation 6) is then calculated between a pair of neurons' kernelized spiking.


(6) $$\begin{array}{l} \text{SI}{\%}_{\text{binless}}=\frac{s_{1}\cdot s_{2}}{||s_{1}||||s_{2}||} \end{array}$$

We compute SI%_binless_ with the time constant ϕ = 5(ms) similar to previous synchronization analysis (Martin et al., 2013). Trial shuffling is also applied for the kernelized binless method. In the Supplementary Figure S2, we explore a variety of hyperparameters for ϕ.

#### 3.4.3 Spatial-attention method

There are a total of five trials for each stimulus in the dataset. Therefore, we train a conditional normalizing flow for each neuron by applying a cross-validation scheme, in which we rotate three trials to form the training set, while the other two form the validation and test sets. The validation set is used for model selection, which ensures that the STAN-Flow will not be overfit to the training data. The spatial-attention module (see Section section 3.2) is learned jointly with the conditional normalizing flow through the loss function (Equation 4). For an arbitrary spike *i* of an arbitrary neuron *n*, and arbitrary stimuli *q*, our modeling process would determine a set of attention weights that determines the importance of each neuron in the neuron population. During our analysis, we concatenate the spatial-attention for each stimulus, then average the spatial-attention weights over all spikes, all neurons, and all runs during evaluation to output a synchronization summary matrix B. The specific calculation is shown in Equation 9.


(7) $$\begin{array}{ll} \beta^{qn}=\underset{i}{\sum }\beta_{i}^{qn}\; & \beta^{qn}\in {\mathbb{R}}^{N} \end{array}$$

(8) $$\begin{array}{ll} B^{n}=\left[\beta^{1n}\cdots \beta^{Qn}\right]\; & B^{n}\in {\mathbb{R}}^{Q\times N} \end{array}$$

(9) $$\begin{array}{l} B=\frac{1}{N}\overset{N}{\underset{n}{\sum }}B^{n} \end{array}$$

Each column of B can be seen as the “strength" of neuron for the *Q* different stimuli. If a subset of neurons with elevated spatial-attention weights for a particular stimulus *q*, it suggests that these neurons are determined to be synchronized by the neural network. An example of this matrix is provided in Supplementary Figure S7.

### 3.5 Classification of neurons into putative PN and LN

Although our ensemble recorded neuronal data does not allow us to identify the neuron types, we follow the classification procedure described in (Lei et al. 2011) to classify PNs and LNs in our spike-sorted units. This classification method relies on the observation that the spontaneous spiking activity of PNs and LNs is different: PNs are more likely to have bursts of spiking activity while LNs fire regularly. It adopts the criterion in (Legendy and Salcman 1985) to detect potential bursts in spontaneous activities (5s) in the full spike train from Poisson Surprise (Poisson*S*) rates, which characterizes the abrupt changes in spiking rates compared to the mean spike rate.


(10) $$\begin{array}{l} \text{Poisson}S=rT-\log\left[\overset{\infty }{\underset{j=n}{\sum }}\frac{{\left(rT\right)}^{j}}{j!}\right] \end{array}$$

The Poisson Surprise rate for a set of spikes is computed from the time span *T* of the set and the mean firing rate *r*, which is the number of spikes *O* in the set divided by *T*. The burst is detected by first finding a pair of successive spikes whose interspike interval (ISI) is less than the mean ISI of the spike train multiplied by a designated coefficient *p*(0 < *p* < 1). Subsequent spikes are added to the pair of spikes to formulate a spike set with the largest possible Poisson Surprise value, and the earliest spikes are pruned from the set if that further increases the Poisson Surprise of the spike set. Finally, the spike set is regarded as a burst if it consists of at least 3 spikes.

With all burst occurrences detected throughout the spike train, we use them to calculate nine burst-related features for a particular neuron (Lei et al., 2011). A logistic regression is finally fitted with the nine burst-related features as covariates to classify the type of neurons as PN or LN.

We train a similar logistic regression model based on the spontaneous spike train obtained from intracellular recordings and staining in (Lei et al. 2011), with a validation accuracy of around 85%. Then, using this logistic regression model, we classify the neurons collected through Section 2. During our initial data analysis, we found that the distribution of the average spike rate in the data from (Lei et al. 2011) differs from that in our spike-sorted data. The difference in distribution resulted in scale differences in the 9 burst-related features (Supplementary Figure S5). To resolve the difference in the features, we applied the following processing steps:

1. We tune the *p* parameter in the procedure for detecting potential bursts to obtain burst-related features on a similar scale. We used *p* = 0.2 while *p* = 0.5 is defaulted in (Lei et al. 2011). The *p* parameter defines the ratio between the mean spike rate (*r*_mean_) and the spike rate of potential burst segments (*r*_burst_) and classifies the segment as a burst when *r*_mean_/*r*_burst_<*p*.
2. We remove three of the nine features for which significant scale differences cannot be resolved by tuning *p*. The six features we used to classify the neuron types are the within-burst maximum spiking frequency, the within-burst number of spikes, the percentage of burst spikes, the burst frequency, the mean Poisson Surprise, and the maximum Poisson Surprise.
3. We apply two different min-max scalers to the training data (Lei et al., 2011) and the testing data (described in Section 2), respectively.

Once the neurons are classified, we supply our predicted labels to human experts to assist in the annotation of true neuron types. We refer the readers to (Lei et al. 2011) for the details regarding the classification method.

### 3.6 Peristimulus time histograms

To see how the individual neurons responded to an odor, we compute a peristimulus time histograms (PSTHs) averaged across five trials for a given odorant stimulus, binned at 20 ms, and smoothed with a Gaussian of 3 standard deviations (Figures 1D, 5B). We generate the response maps of the odor-evoked population response by Equation 11. Here, we *z*-scored the PSTHs for each neuron by subtracting its average baseline firing rate (measured 200 ms before a stimulus onset) and dividing by the standard deviation of the baseline. The response maps indicated changes in firing rate in units of the standard deviation of spontaneous activity. The (Figures 1D, 5B) are used for visualization purposes only.


(11) $$\begin{array}{l} {\text{PSTH}}_{z\left(t\right)}=\left(\text{PSTH}-\mu_{\text{baseline}}\right)/\sigma_{\text{baseline}} \end{array}$$

### 3.7 Response index

The Response Index was computed in our study to investigate the response of different units to the odor stimuli and to assess the similarity of generated and real spike trains under different stimuli for each unit (Riffell et al., 2009a). The response index is calculated as follows:


$$\begin{array}{l} {\text{RI}}_{\text{odor}}=\frac{\left(r_{\text{odor}}-r_{\text{control}}\right)-r_{\text{mean}}}{SD} \end{array}$$

where the *r*_odor_ is the firing rate of a specific odor; *r*_control_ is the firing rate of control (mineral oil); *r*_mean_ is the mean firing rate averaged over all stimuli, and *SD* is the standard deviation of the firing rate across all stimuli. Firing rate in this RI computation specifically means the average firing rate over the stimulation period (0–600 ms after the onset of stimuli). The response indexes of other preparations are shown in Supplementary Figure S3.

### 3.8 Euclidean distance

Euclidean distance is applied in our study to understand the difference between spatial-attention vectors of different stimuli. For two vectors $v_{1},v_{2}\in \mathbb{R}$ for an arbitrary dimension *d*, with *v*_1*i*_, *v*_2*i*_ denoting the *i*^*th*^ element of the corresponding vector, the Euclidean distance is defined as


(12) $$\begin{array}{l} \text{Euclidean distance}=\sqrt{\overset{d}{\underset{i=1}{\sum }}\left(v_{1i}-v_{2i}\right)} \end{array}$$

## 4 Results

By leveraging well-known biological principles of the spiking and interaction between neurons in the primary olfactory center, we designed STAN-Flow to model the fundamental neuron spiking mechanism and neuron interactions in the AL (Figure 1F). We validate STAN-Flow from three different perspectives: (1) Can it generate realistic spike trains that replicate the statistical distribution of recorded data? (2) Can it distinguish between behaviorally relevant and irrelevant stimuli using spatial-attention weights that reflect neural synchronization patterns? (3) Can it infer interactions among distinct neuronal subtypes in the AL? After validating that the STAN-Flow effectively learns the spiking mechanism and network dynamics, we apply it to investigate whether synchronization is affected when component odorant concentrations diverge from those found in natural, behaviorally significant mixtures (e.g., floral blends that drive foraging behavior).

### 4.1 Antennal lobe network dynamics and spike train generations

One major aspect of validating the STAN-Flow architecture is examining how similar the generated spike trains are to the empirical neuron recordings. In Figure 2A, we present five trials of the spike train of a specific unit in response to a behavioral blend odor (B19) and a generated spike train given this odor. Visually, the generated spike train (magenta, Figure 2A), which is generated conditioned on data in the test set, realistically captures the temporal dynamics of the spiking pattern of spontaneous baseline activity, stimulation period, and post-stimulation corresponding to the empirical spike train.

We conclude that there are no differences in the empirical and generated distribution of ISI (Kolmogorov–Smirnov (KS) Test^1^, *K* = 0.08, *p* = 0.98; Two One-Sided *T*-Test (TOST) for distribution mean^2^ (Lakens, 2017) (*t* = 1.29, *p* = 0.09). We also found no significant differences in the average firing rate of a neuron for empirical and generated spike trains (two-sided *t*-test, *t* = 0.65, df=8, *p* = 0.53), indicating that the generated spikes are similar to the empirical ones (see Figures 2A1, A2).

We also examine the generated spike trains across an ensemble. We present the empirical and generated spike trains of 14 units stimulated with behavioral odor B_16_ and control, respectively (Figures 2B1, B2). Surprisingly, the response index (RI; Figures 2C1, C2, Supplementary Figure S3) shows strong correspondence between empirical and STAN-Flow-generated spike trains across all neurons and stimuli. This close match indicates that STAN-Flow effectively captures the average spiking patterns observed in empirical recordings. The consistency of this alignment across multiple neurons and stimuli further highlights STAN-Flow's state-of-the-art capability in modeling the spiking dynamics of neurons within the antennal lobe (AL) region.

### 4.2 Spatial-attention weights classify stimuli

The AL relies on different cell pairs synchronizing with one another, or a specific subset of critical neurons, to encode both behavioral and non-behavioral stimuli (Riffell et al., 2009a; Lei et al., 2016). Our method captures this synchronization mechanism through the spatial attention module. The spatial-attention module assigns an attention weight to each of the units of the spike train input. Each row of the synchronization summary matrix B (see Equation 9) represents a stimulus, and the columns represent the relative importance of each unit. The spatial-attention method takes all neurons into account during the modeling of spike trains, thus offering the ability to characterize the synchronization of multiple neurons beyond pairwise analyses.

We first apply t-distributed Stochastic Neighbor Embedding (TSNE) to reduce the dimensionality of B. Our result demonstrates that the spatial-attention weights distinctly separate the stimuli into two clusters: one includes all the stimuli mixtures that contain all the behavioral components (green), and the other cluster includes the individual odor molecules (purple) as well as mixtures containing non-behavioral components (light purple) (Figure 3A, Supplementary Figure S4). Two exceptions are grouped with the behavioral relevant stimuli space: the single odor (Benzylalcohol: O_2_) and non-behavioral blend (NB_10_ (mixture containing (O_1_ and O_2_): Riffell et al., 2009a). These results suggest that O_1_ and O_2_ could be essential to the behavioral responsiveness of complex odors. To ensure reproducibility of our result, we retrained STAN-Flow with the same configuration using 100 different random initializations, then applied a 2-class K-means algorithm (behavioral vs. non-behavioral), STA yields around 80 (±4.5)% classification accuracy.

We next compute the pair-wise synchronization matrix for two previously known methods: the Ensemble Synchronization Index (ESI) (Riffell et al., 2009a) and the Kernelized binless method (KB) (Martin et al., 2013). We first extract the upper triangular synchronization matrix of ESI and KB, then apply TSNE to reduce its dimension. We do not observe obvious patterns of clustering in these methods (Figures 3B, C). Compared to the STA results (STAN-Flow trained with 100 different initializations), both of these methods yield around 20% less accuracy (One-sided *z*-test; ESI: *z* = 4.67, *p*-value < 0.01; KB: *z* = 4.67, *p*-value < 0.01), indicating that STA consistently outperforms ESI and KB (Figure 3E). As ESI and KB were previously only applied to populations of projection neurons (Riffell et al., 2009a; Martin et al., 2013), we conjectured that ESI and KB failed to separate the stimulus types in this neural population because both LNs and PNs are present. This enhanced performance in separating the behavioral and non-behavioral stimuli when different types of neurons exist in the neuron population propels us to understand how the spatial-attention models the interaction of different neuron categories in the AL.

We also compare the effect of different window sizes [Δ] in extracting neural synchronization. Similar to our comparison before, we apply TSNE on the STA generated from models trained with different window sizes, then apply K-means on the dimension-reduced STA and compute K-means accuracy (Figure 3D, highlighted with light green). We found that while a 20 ms window performs the best, synchronization can be captured with a window size as low as 5 ms. STAN-flow trained a wide range of window sizes continue to outperform traditional methods, suggesting that STA is a robust tool to measure synchronization at any level.

### 4.3 Detecting the interaction between PNs and LNs

The antennal lobe consists of broadly two types of neurons, PNs and LNs. As (Lei et al. 2004, 2016) and (Tanaka et al. 2009) suggested, LNs modulate the PNs synchronization, and the PNs synchronize among themselves (Martin et al., 2013) to encode behavioral and non-behavioral stimuli. While STAN-Flow is trained without the knowledge of neuron types, we investigate if STAN-Flow can recover the interaction between LN and PNs through modeling the spiking process. Hence, we need to label the neurons to their corresponding neuron types. However, with the electrophysiological recordings in the AL with the current method, we cannot morphologically classify PNs and LNs. To classify the neurons, we utilize previous results that found the PNs and LNs have distinctively different spontaneous patterns of spiking activities (Figures 4A, B) (Lei et al., 2011). The PNs burst from time to time during spontaneous baseline firing, while the LNs spike regularly. Based on this observation, (Lei et al. 2011) developed a simple method to classify the neurons based on their spontaneous spiking dynamics.

We refer readers of the details of this classification process to Section 3.5. The classification accuracy computed against expert label was 78% while the validation accuracy using data from (Lei et al. 2011) is about 75%. Since there could be more PNs than LNs in the AL region of the moth (Homberg et al., 1988; Reisenman et al., 2011), we also compute the recall (0.73) and precision (0.73), showing that the classification method obtained a reliable result under this imbalance classification scenario.

With the neuron labels, we continue to study the essential question in this analysis to understand the interaction between the PNs and LNs. To better understand the functional roles of local neurons (LNs) and projection neurons (PNs), we trained our model on the full population of recorded neurons, then separately visualized the synchronization learned by STA of PNs (Figures 4C, C1) and LNs (Figures 4D, D1) using 2D TSNE. The PNs' embeddings showed clear clustering of behavioral vs. non-behavioral stimuli (highlighted with a gray background rectangle, while the LNs lacked any discernible pattern. When we trained the model using only the annotated PNs or LNs, the PNs alone still exhibited visible clusters corresponding to behavioral stimuli, with partial overlap from non-behavioral stimuli, suggesting inherent synchronization among PNs. One notable feature is that the individual odors are distinctly cluster (Figure 4D). Importantly, training on PNs extracted from the full model resulted in a mean classification accuracy approximately 12.5% higher than training on PNs alone (one-sided *z*-test, *z* = 20.77, *p*-value < 0.001). This enhancement suggests that the optimal stimulus separability may be modulated by LNs, which could suppress responses to non-behavioral stimuli. Collectively, these results highlight the critical role of LNs in refining PN synchronization and emphasize their importance in the encoding of behavioral stimuli within the AL and the STAN-Flow model.

Our previous result has highlighted that LNs are critical for odor classification, but could there be a core neuronal unit in an ensemble that accounts for the segregation of behavioral and non-behavioral stimuli? As we showed that spatial-attention consistently clusters the behavioral and non-behavioral odors, we now test the K-means clustering accuracy by removing each unit in an ensemble. We found one LN significantly lower than the classification accuracy (86% before removal vs. 78% after removal). This neuron responds to both behavioral compounds and non-behavioral compounds (Figure 2C1), unit 14. In addition to single neuron analyses, we removed a combination of up to 3 neurons (data not shown) in an ensemble. We found a specific combination including the LN (Figure 2C1, unit 14; Supplementary Figure 3) and two other PNs (units 2 and 5, Figure 2C1; Supplementary Figure 6) will lower the clustering accuracy to 52%. These PNs responded to the majority of the behavioral compounds (broadly tuned). These results also further indicate that broadly tuned LNs could be core neurons for odor classification (Wilson et al., 2004; Olsen et al., 2010) in combination with the PNs, which are responsive to the behavioral compound (Figure 2C1).

### 4.4 Altering the excitatory drive attenuates synchronization

STAN-Flow preserves the fundamental spiking dynamics and functional architecture of the AL by capturing the synchronization patterns of projection neurons to the behavioral mixture, including behaviorally relevant odor diluted by several magnitudes (Figure 3A, odors (B_20_, B_22_, and B_22_) in the behavioral clusters). To test the robustness of our model in capturing this synchrony, we increased the concentration of a single component, benzaldehyde (O_1_), within the behavioral odor mixture B_23_ to 10-fold (B_24_), 100-fold (B_25_), and 1,000-fold (B_26_). Visually, the stimulus concentration-dependent response was prominent. The PSTHs show the sustained activity after the stimulation, forming a plateau-like PSTH to the increased O_1_ ratio as compared to the original B_23_, indicating the weak or delayed inhibitory input to this neuron (Figures 5A, B, E). These dose-dependent increases in the O_1_ ratio led to a transient suppression in neuronal response patterns, which may be due to the inhibition of inhibitory neurons involved in encoding odor identity/intensity (Figures 5B, E).

We retrained STAN-Flow using neural activity. We then repeated the TSNE analysis on the summary matrix (see Figure 5C). The TSNE clustered individual components toward the top-right quadrant, grouped the behavioral mixture B_23_ variants with elevated O_1_ concentrations centrally, and positioned the control stimuli to the far left. Also, the response index of the generated heatmap (Figure 5D, Supplementary Figure S6) is similar to the empirical response index.

To evaluate how ensemble synchronization changes with increased O_1_ ratio in the mixture, we calculated the dissimilarity of odor-evoked synchrony patterns using a normalized dissimilarity index (normalized by the mean, moth = 3, units = 43). This analysis compared B_23_ with 10 × , 100 × , 1000 × increase of O_1_ (B_24_- B_26_) and also with individual odorants. As shown in Figure 5F, there is an increasing trend of the dissimilarity with B_23_, but it is not statistically significant among the group (one-way ANOVA, *F* = 0.642, *p* = 0.673). These findings could suggest that elevated O_1_ concentrations in the behavioral components may shift the neural representation toward a non-behavioral classification.

Our results underscore the role of synchrony in the AL as a critical coding mechanism for odor classification. This synchrony is finely tuned to both the identity and the concentration of odor components, enabling flexible and accurate recognition of behaviorally relevant odors in dynamic natural environments.

## 5 Discussion

Here in the current study, we elucidated the effects of network dynamics in the antennal lobe (AL) to classify behavioral and non-behavioral relevant odors. We developed the spatial-temporal attention normalizing flow (STAN-Flow), an accurate computational model representing the spiking ensemble dynamics of the AL. We adopt this model to extend the characterization of the AL network beyond the experimental possibilities. The model effectively reproduced the AL responses' key features concerning the odor classification, through detailed neuron-level interactions. This model also agrees with the previous results that local interneurons play critical roles in the temporal encoding of odor stimuli, enabling the classification of odors into behavioral and non-behavioral stimuli. Shifting the concentration of one of the behavioral components in the odor mixture—by altering the excitation/inhibition balance in the AL—causes the neuronal representation of this stimulus to change. This computational model can be easily modified to be applied in various fields for accurately modeling and reliably interpreting complex interactions for biological and non-biological systems.

### 5.1 Neurophysiological computation in the AL

The AL is one of the most extensively studied neural structures in the insect brain, particularly in terms of its detailed cellular and circuit-level architectures for sensory encoding. Over the last two decades, behavioral, physiological, and modeling research have made significant strides in understanding the circuit basis of processing complex odor mixtures, their intensities, and their relationship to odor classification. Understanding the role of the AL in odor perception has been the focus of a variety of theoretical and computational models. The dynamic and complex stimuli necessitate utilizing the computational models to extract the features of interest from the spike trains (Triplett and Goodhill, 2019). The STAN-Flow developed in this study serves this purpose: its flexibility simulates the spike train generation process and successfully discriminates and classifies complex odors. This model could be beneficial in identifying future odors, whether or not they could be relevant to insects, predicting the population response, and simulating the spike trains of the neurons.

This computational model can cluster the odors into behaviorally relevant and non-behaviorally relevant groups (Figures 3, 5). Our approach enhances clustering into behavioral and non-behavioral odor stimuli, and it is highly efficient. It could potentially facilitate further processing in the higher brain centers such as the lateral horn (LH) (Lazar et al., 2023; Strutz et al., 2014). Given the extensive knowledge of neuromorphic processing in downstream neurons from the AL and its circuits, it remains unknown to date how this spatiotemporal information is processed within the AL and in higher brain centers.

### 5.2 Local interneurons necessitate the PNs' synchronous activity

Olfactory information is encoded as spatial-temporal patterns in the neural population of the AL, and through the activity of different cell types, such as LNs and PNs. A benefit of the model and resulting analyses is a dissection of the contribution of different cell types in how the complex odor stimuli are classified into behavioral and non-behavioral (Figures 3, 5). However, the glomerular processing of the behaviorally relevant odor is putatively identified in the anterior lateral regions of ordinary glomeruli in the AL investigated through measuring the calcium activity of sensory neurons in the moth (Figure 1 in Bisch-Knaden et al., 2018; Bisch-Knaden et al., 2022). It appears that the region processing the three components of behaviorally relevant odors is colocalized in a similar position within the ordinary glomeruli in the AL. However, the PNs and Lns involved in these colocalized glomeruli are not known, even though diverse morphological PNs and LNs are reported in different insect species (Kymre et al., 2021; Matsumoto and Hildebrand, 1981; Kuebler et al., 2012; Reisenman et al., 2011; Chu et al., 2020). Future experiments with simultaneous recording from the selective recordings will answer the neural circuit involving such behavioral valence. Unlike some specific glomeruli (particularly pheromone processing glomeruli), which may not require specific connectivity between the glomeruli (or sparse connection) for the PN response, some floral odor processing glomeruli (ordinary glomeruli) and PNs arising from these glomeruli may involve the LNs that have heterogeneous branching pattern in these glomeruli and could channel to the behavioral relevance odor. Of 1,100 PNs, we were only able to identify 78% of the PNs. Even with extensive sampling of LNs and PNs through intracellular recordings and staining in the other moth species (*Helicoverpa armigera*: out of 176 reported neurons, 61% are PNs (Kymre et al., 2021). Our method also relies on validation techniques from intracellular recordings of neuron categories in the AL of the *Manduca sexta* (Lei et al., 2011), and the number appears to be comparable. We have not subcategorized the different subclasses of LNs and PNs due to a lack of staining data. The existence of subcategories of the PNs with the distinct physiogical properties during the spontaneous (Capurro et al., 2014) and after the stimulus offset and explain why we cannot achieve 100 percent accuracy. In line with other studies on response properties of PNs on the fruit flies (Wilson et al., 2004). The majority of the PN responses are dynamic under different odors; some are activated, and some are inhibited with no responses (Figure 2C, Supplementary Figure S3) due to the interaction of the olfactory sensory neurons (OSNs) and LNs. The excitatory feedforward information from OSNs activates PNs, which in turn indirectly activate LNs.

The inhibitory LN connecting the defined subsets of the glomeruli could play a crucial role in understanding perceptual constancy in the olfactory circuits, especially in understanding the synchronized activity of the PNs in response to the behaviorally relevant mixture. Previous studies have shown that pharmacological receptor antagonists, targeting the GABA receptors, abolished the synchronized activity of AL neurons and affected the olfactory behavior of the moths (Lei et al., 2011; Riffell et al., 2014). The LNs modulate the temporal patterns of PNs' spiking responses, resulting in odor-evoked activity which can enhance the synchrony of sister PNs within the same glomerulus as well as the synchrony of co-activated PNs from the other glomeruli (Lei et al., 2002; Martin et al., 2013). However, the structural connectivity information is still lacking in *Manduca sexta*, which limits our understanding of the synaptic-level connections between neurons. Electron microscopical studies in fruit flies have shown that the OSNs contribute 75% of the synaptic input to PNs, and the remaining 25% is contributed by the LNs (Tobin et al., 2017). It is possible that the LN that makes specific synaptic connections with a given glomeruli could provide the postsynaptic inhibition to the PNs that are processing the non-behavioral relevant odors and receiving information from the subset of behaviorally relevant neurons. This can be ecologically relevant in inhibiting the input of the non-behavioral stimulus pathway, as it may be the background. In various organisms, such as moths and fruit flies, the LNs contain both pre- and postsynaptic synapses, and the density of these synapses is biased across different glomeruli. This bias could eventually affect the extent of lateral inhibition processing in the mixture (Hong and Wilson, 2015; Silbering and Galizia, 2007).

### 5.3 Concentration varying effects on AL network dynamics

Navigating through the complex and dynamic olfactory environment, the moth is challenged with fluctuating odor concentrations. The moth should evaluate the odor and its intensity. Behaviorally, *Manduca* has been shown that a subset of the behaviorally relevant odorants is processed in a quick (< 500 ms) and reliable manner (Riffell et al., 2009b). Suppose if the ratio of one of these compounds is changed in the behavioral mixture, then they could be evaluated as a different floral compounds and the neural population responses could vary for those altered composition and could be clustered outside the neighborhood of the behavioral mixture (Figures 3A, 5C) (Riffell et al., 2009a, 2014). The behavioral compound such as Benzaldehye (O_1_) which is one of the important constituents in the behavioral mixture is clustered with the non-behavioral mixture (NB_10_) that contains Benzaldehye (O_1_) and Benylalcohol (O_2_) could play a significant role in the discrimination of odors in behavioral or non-behavioral and thus affect the olfactory navigation.

The decreasing intensity of the floral mixture, diluted upto 10,000-fold clustered with the behavioral mixture in the previous study (Riffell et al., 2009a) and this study (Figure 3) could be due to gain control of the LNs (Sachse and Galizia, 2002). Altering the ratio of (O_1_) in the behavioral floral mixture (B_23_), we noticed that different ratios are clustered outside the neighborhood of the B_23_. The concentration of the same odor may have different or even opposite values (Semmelhack and Wang, 2009), and this odor could modify the quality of the odor (Laing et al., 2003). At the presentation of increased ratio of an odorant in the behavioral floral odor, the LNs may modulate (Supplementary Figure S5) and fine-tune the PNs for synchronization, therefore clustering the neural responses into the behavioral and non-behavioral components (Clifford and Riffell, 2013). However, upon increasing the ratio of one of the components, the GABAergic neurons may be recruited (Laurent, 2002) thus the presynaptic inhibition of sensory neurons by these neurons (disinhibition) could play a role in odor discrimination. However, how the modification to the odor panel influences the molecular mechanism or alters the plasticity within the AL remains unknown. In our experiments, we present moths to an elevated ratio of benzaldehyde within the natural concentration range. In *Datura wrightii* flowers, benzaldehyde is emitted at about 0.23 ng/h (Riffell et al., 2009a). Increasing this concentration by the 1,000-fold (230 ng/h) still falls below the natural emission rate observed in other putative hawkmoth-visited flower, such as *Petunia hybrida*: 8,000 ng/h (Boatright et al., 2004) and in blossoms and branches of Crabapple (*Malus sp:* 5,580 ng/h) (Baghi et al., 2012). These comparisons indicate that the moths in our study were stimulated within ecologically relevant, naturally occurring ranges.

### 5.4 Limitations

There remain a few limitations in the STAN-Flow architectures and related analysis. One main limitation is the interpretability of the neural networks. Although through post hoc analysis, we showed that the spatial-attention weights can be interpreted to a great extent, there are limited theoretical analyses on the attention module to guarantee the interpretability of the attention weights. There are, however, ways to improve the credibility of the result: one can cross-validate with established results to check if the interpretability matches expectations, as we did with PN and LN classification. In addition, one could repeat the experiments many times (e.g., 100 different initializations) and across subjects to verify the consistency of the result. Another drawback of this current architecture is its scalability. The current training scheme of STAN-Flow for performing group-wise synchronization analysis requires neuron-specific STAN-Flow. While the number of models scales linearly with the number of interacting neurons, it remains challenging to analyze a larger population of neurons at a greater scale. Given different modeling scenarios, however, this could be resolved by a more intricate STAN-Flow that can be modified to model synchronization hierarchically when dealing with neurons from different regions; it could also be easily adapted to model a multidimensional time series instead of modeling each neuron at a time. Finally, the recent rise of diffusion generative modeling techniques can be applied to improve the normalizing flow (Song et al., 2020; Hasan et al., 2023). There also exist ways to directly connect the interaction between neurons with the generative component. For example, the interaction between neurons can be modeled as interacting particle systems characterized by a McKean–Vlasov diffusion, which can be applied as a latent process to significantly enhance the interpretability of the machine learning system (Yang et al., 2024). Despite these limitations, our machine learning approach offers a broader impact.

### 5.5 Significance and broader impact

Our STAN-Flow explores a new, computational avenue to study interactions within neural systems. The interaction of multiple sensory systems is common in many biological organisms, allowing them to respond swiftly and efficiently to complex and dynamic environments. For instance, in other animals and humans, multiple peripheral, central, and motor systems work in concert to produce coordinated behaviors. One prime application of the spatial-temporal attention module, a significant focus of our ongoing research, is the modeling of multisensory binding in the brain that is funneled downstream via the descending neurons to the motor program. The neural mechanism of integrating multisensory information in the brain is not well understood, and how it drives the motor program is still under investigation. The motor programs involve the coordinated activity of individuals and groups of flight muscles that interact dynamically to produce agile movements and abrupt changes in behavior (Putney et al., 2019). Despite extensive research, the interactive mechanisms that govern this muscle dynamics remain largely unknown (Putney et al., 2023). The spatial-temporal attention module has the potential to uncover these mechanisms by providing a framework that captures the intricate timing and spatial relationships involved in motor coordination.

Another significant benefit of the STAN-Flow model is its deep generative component. Biological interactions are inherently stochastic and often do not conform to traditional statistical distributions such as the Poisson or Gaussian distributions (Lindner, 2006; Deger et al., 2012). This stochastic nature presents challenges for conventional modeling approaches that rely on these distributions. The introduction of a flexible generative model through neural networks, as seen in STAN-Flow, allows for more accurate modeling of natural phenomena with fewer assumptions about the underlying distributions. This flexibility is particularly important when exploring complex biological interactions that may be high-dimensional and highly non-linear. By leveraging the power of neural networks, STAN-Flow can capture the rich and varied nature of biological data, providing deeper insights into the interactions between multiple brain regions or systems.

STAN-Flow's success in modeling the antennal lobe region has illuminated the potential of combining attention mechanisms with deep generative neural networks to understand the complex interactive relationships between organisms and their environments. The ability of STAN-Flow to accurately model the dynamic and non-linear interactions in the AL region suggests that similar approaches could be applied to both biological and non-biological systems. This attention module could be applied to other sensory systems, such as the visual and auditory systems, for stimulus discrimination (Wilsch et al., 2020). However, whether one of the systems favors one of the modules (spatial or temporal) is elusive. This opens up new avenues for research in understanding how different neural systems interact and adapt to their environments, ultimately contributing to a more comprehensive understanding of biological complexity and adaptability. Apart from its biological context, STAN-Flow can also be applied in the digital field (Gao et al., 2023). The STAN-Flow can be naturally applied to related tasks such as analyzing videos and speech by summarizing the interaction of different graphic regions and condensing the importance of different periods of videos. By enforcing a set of constraints on the attention weights, it is also possible to extend the module to track objects through space and time. As many climatic phenomena also originate from interactions of local climate (Saupe et al., 2019), STAN-Flow also provides a generative predictive algorithm that allows explicit interaction between local climates to forecast weathering trends in the future. In general, the flexible, semi-interpretable neural network structure of STAN-Flow offers a wide range of applications that can help inform scientists with high-order interactions between groups or individuals over time.

## Data Availability

The original contributions presented in the study are included in the article/Supplementary material, further inquiries can be directed to the corresponding author.

## Footnotes

^1^The KS-Test will reject the null hypothesis if two distributions are statistically different.

^2^Also known as the test of equivalence for two independent samples; the TOST will reject the null hypothesis if the means of the two distributions are within the ±Δ region. We choose Δ = 2 ms.
