# Supplementary material for: Neuron synchronization analyzed through spatial-temporal attention
Source: Front Comput Neurosci. 2025 Oct 16;19:1655462. doi: 10.3389/fncom.2025.1655462 (PMC12571808; doi:10.3389/fncom.2025.1655462)
Supplement: Supplementary file 1 [file Supplementary_file_1.pdf]

Supplementary Material

1 HYPERPARAMETERS OF STAN-FLOW

Throughout the study we used a window size $[\Delta] = 20$ msec, our data has a resolution of 1 msec, so 20 msec resulted in a dimension with size 20. We note that 20msec was chosen based on empirical experiments, as we will demonstrate in section . The LSTM encoder has 2 layers of LSTM units, each has 4 hidden dimension. The output dimensions of the embedding layers for the attention module are 10. The stimuli are one-hot encoded. For the normalizing flow, we applied the RealNVP architecture (Dinh et al., 2016) to construct a normalizing flow with 4 coupling layer blocks, each with a hidden dimension size of 64. The activation function for the s_net is *tanh* and the activation for t_net is *softplus*. To train STAN-Flow, we used an AdamW optimizer with a learning rate of 1e-5.

2 ADDITIONAL RESULT

In this section of the supporting information, we include additional results, including testing different parameters for pairwise synchronization methods, Response index matrix for other preparations, testing spatial attention's performance across preparations, and exploring the PN, LN interaction with stimuli that has altered odor ratios.

2.1 Testing different parameters for Ensemble Synchronization and Kernel Binless Methods

In this subsection, we test the Ensemble Synchronization index and kernel binless methods across multiple preparations with a wide range of hyperparameters. We ran similar 2D TSNE analyses as in section . Specifically in Fig S1, we compute ensemble synchronization over two different periods (0-500 msec, 0-1000 msec after onset of stimuli), and different binsize (5, 10, 20 msec). Limited clustering can be observed across different subjects and different parameters for the Ensemble synchronization index method.

For the Kernel binless method, we tested $\tau \in [2, 3, 4, 5, 6 \text{ msec}]$ used to tune the exponential kernel. There is, however, no obvious separation between the behavioral and non-behavioral stimuli (Fig S2).

2.2 RI With Neuron Type

We show the STAN-Flow generated and real Response index (RI) in Fig S3. The response index matrices between emperical and real spike trains are similar, indicating that the STAN-Flow's generative performance is consistent across preparations.

2.3 Spatial attention performance on other preparations

We also conducted similar analyses in section across different preparations as well. Although the spatial attention does not cluster for these preparations, we show that the behavioral and non-behavioral stimuli are still linearly separable (see Fig S4). We used a logistic regression to classify and obtained an average training accuracy of 79% over 4 subjects. The lack of clustering through TSNE might be due to strong trial to trial variation, leading to noisy signals in the $\mathcal{B}$ matrix.

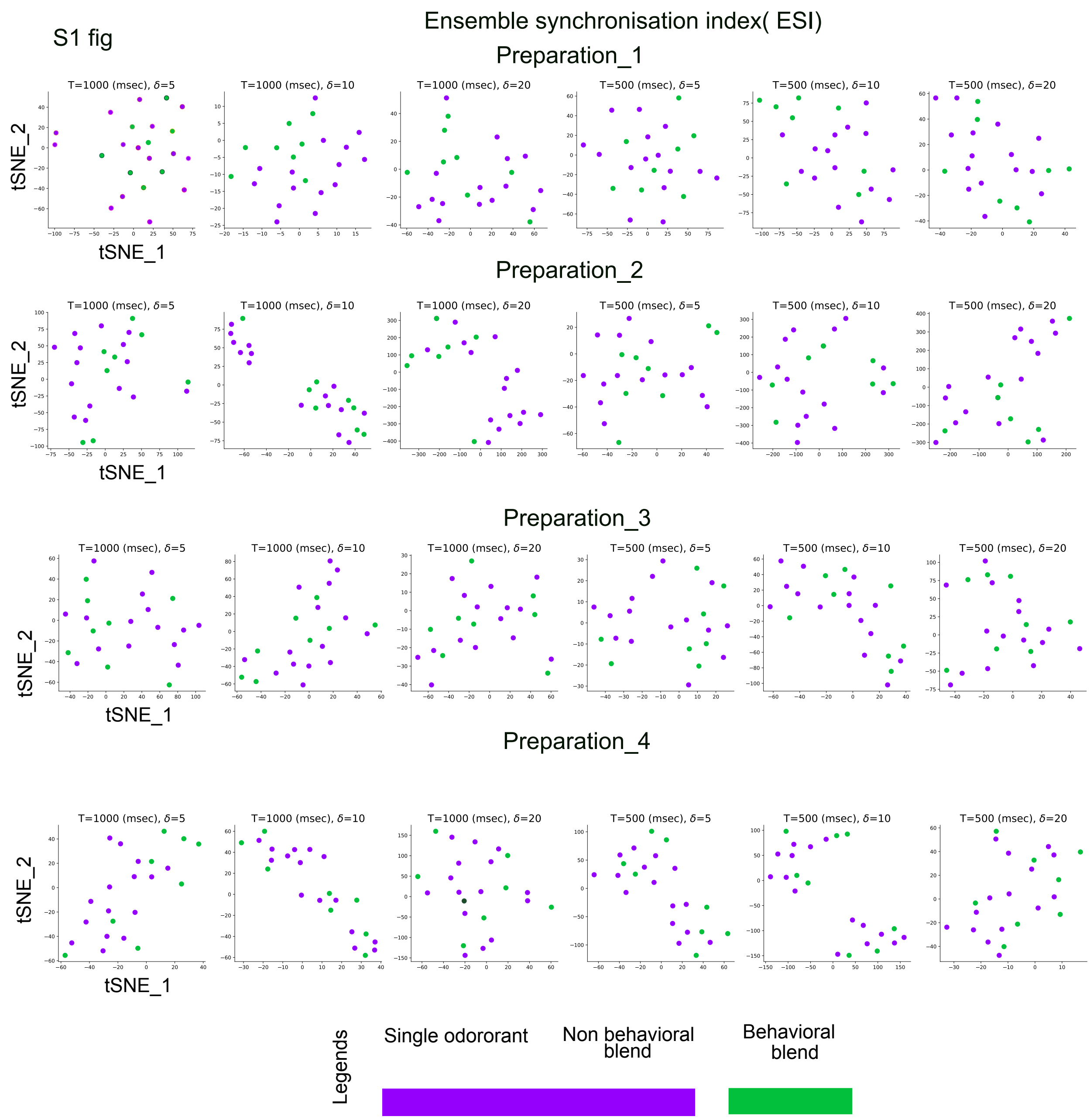

Figure S1. Two dimension TSNE analyses with the upper triangular matrices of the pairwise ensemble synchronization methods applied with different parameters on different preparations. T denotes the length of the stimulation segment that is analyzed, and δ denotes the binsize used in that specific computation of the ensemble synchronization index.

2.4 Burst Features for Classifying Neuron Types

We show that the 6 parameters chosen to classify neuron types has similar distributions to those in Lei et al. (2011) after our processing pipeline.

2.5 Interaction between PNs and LNs with altered stimuli ratio

We show the clustering result of different neuron types for stimuli with altered ratios of Benzaldehye (O_1) in behavioral mixture (B_{23}) . Fig S6A and S6B shows the clustering of the different increased ratio of Benzaldehye (O_1) with behavioral compound. We also showcase the RI of this experiment.

3 SPATIAL ATTENTION WEIGHT

In Fig S7, we present an example of Spatial attention weight, which can be understood as a representation of the neural synchronization learned by the neural network.

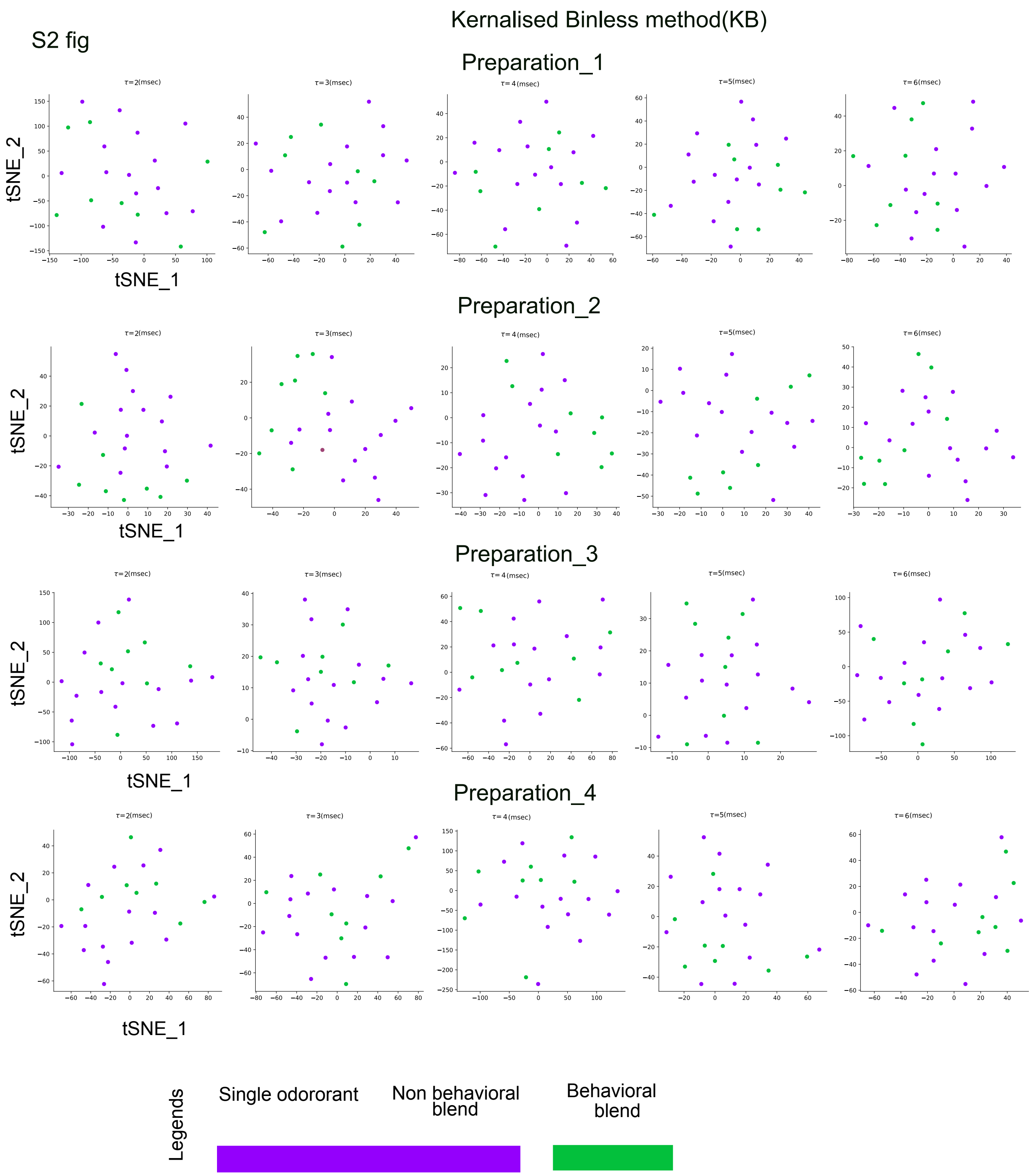

Figure S2. Two dimension TSNE analyses with the upper triangular matrices of the pairwise kernel binless methods applied with different parameters on different preparations (1-4). τ denotes the time constant parameter of the exponential kernel applied in the kernel binless method.

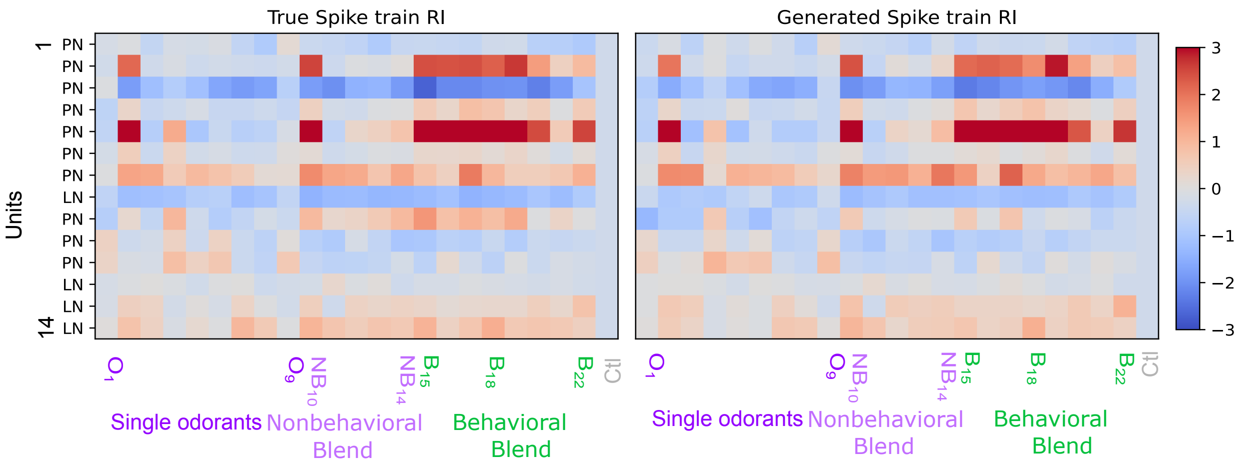

Figure S3. Response index between real and Generated spike. Y axis is indicated with the neuron categories and z axis with different individual odorants, Nonbehavioral blend and behavioral blend

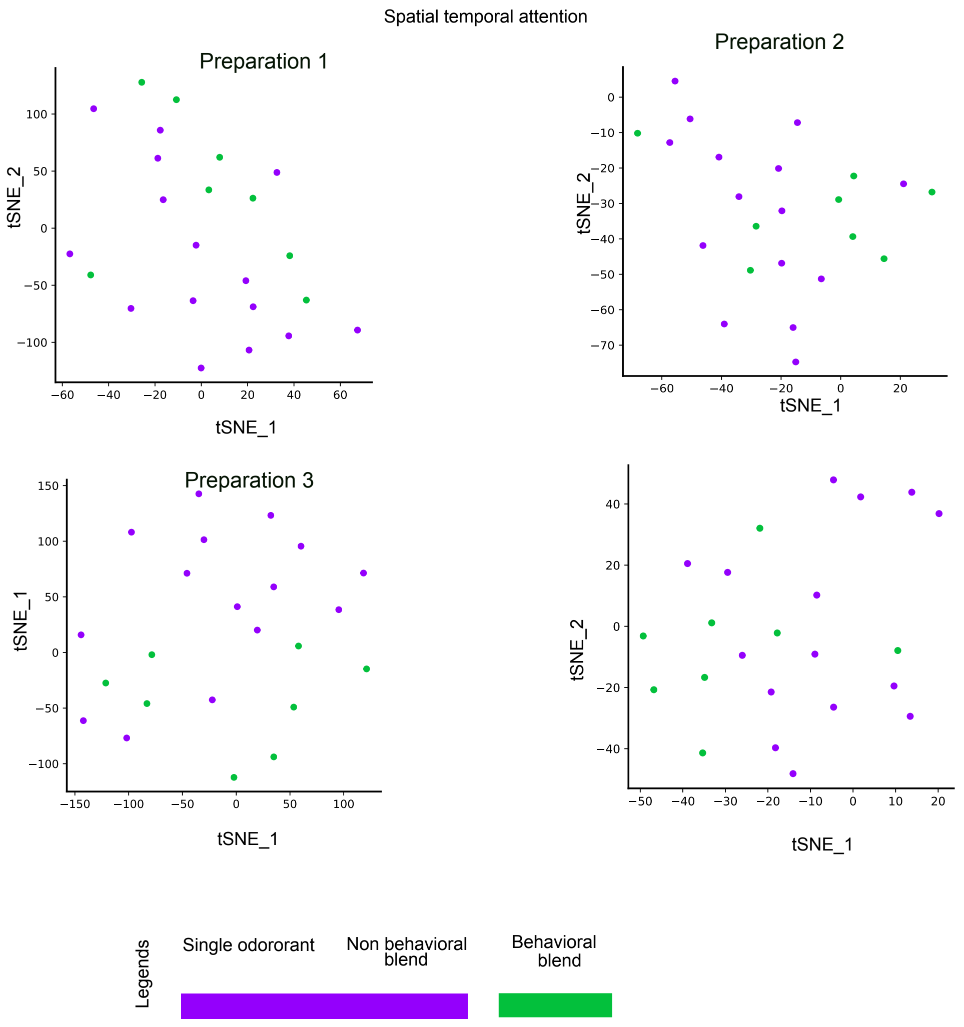

Figure S4. Spatial attention weights separate the behavioral stimuli (green) from the non-behavioral stimuli (purple).

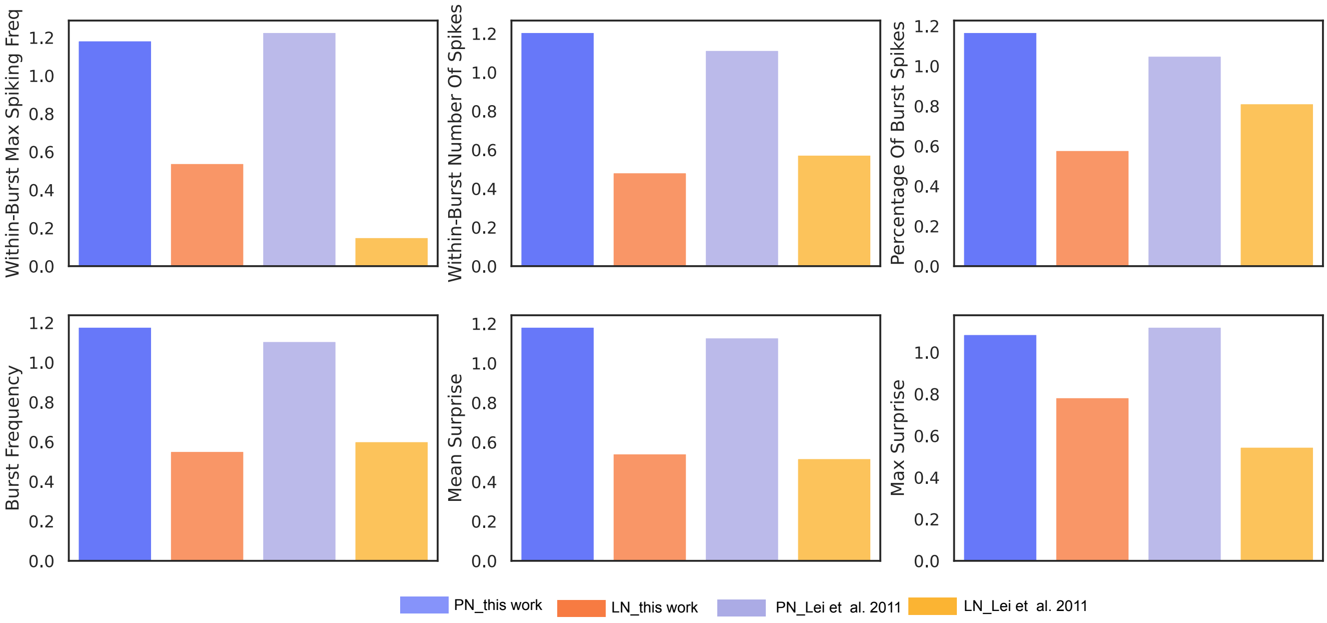

Figure S5. The 6 electrophysiological parameters used to classify neuron types in our study.

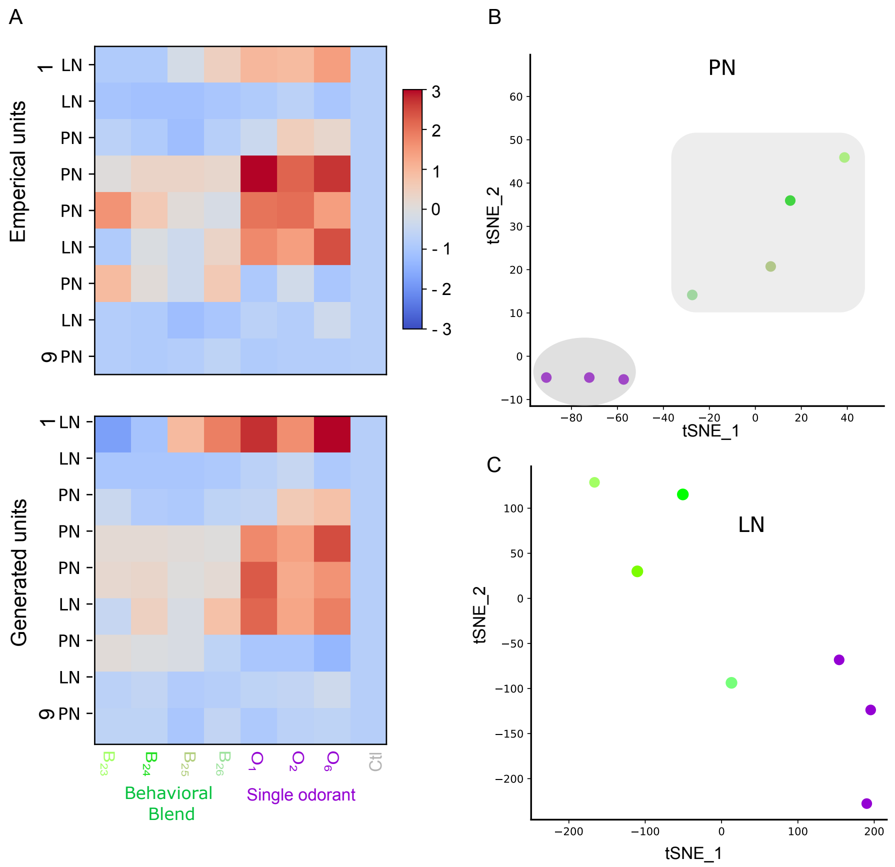

Figure S6. Clustering of the increased concentration of Benzaldehye (O_1) with behavioral compound. (A) Heat map showing the response index of increased O_1 ratio in the behavioral blend (B_{23}). (B)The TSNE plot of the PNs (B) demonstrates the clustering of the increased ratio of O_1 in behavioral blend (B_{23}). The shaded brown rectangle represents the clustering of behavioral blend and shaded circle represents individual odorants with the projection neurons (PNs). (C) The TSNE plot of the LNs.

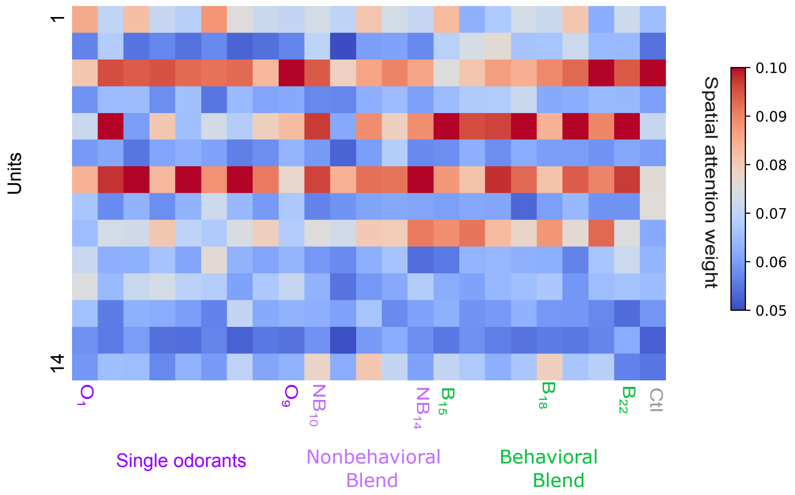

Figure S7. An example of Spatial attention weight heat map

REFERENCES

Dinh, L., Sohl-Dickstein, J., and Bengio, S. (2016). Density estimation using real nvp. *arXiv preprint* arXiv:1605.08803

Lei, H., Reisenman, C. E., Wilson, C. H., Gabbur, P., and Hildebrand, J. G. (2011). Spiking patterns and their functional implications in the antennal lobe of the tobacco hornworm manduca sexta. *PLoS One* 6
